# Semiclassical Theory of Multistage Nonequilibrium Electron Transfer in Macromolecular Compounds in Polar Media with Several Relaxation Timescales

**DOI:** 10.3390/ijms232415793

**Published:** 2022-12-13

**Authors:** Serguei V. Feskov

**Affiliations:** Institute of Mathematics and Information Technologies, Volgograd State University, Universitetskiy Prosp., 100, 400062 Volgograd, Russia; serguei.feskov@volsu.ru

**Keywords:** electron transfer, macromolecular compounds, photochemistry, ultrafast reactions, nonequilibrium processes

## Abstract

Many specific features of ultrafast electron transfer (ET) reactions in macromolecular compounds can be attributed to nonequilibrium configurations of intramolecular vibrational degrees of freedom and the environment. In photoinduced ET, nonequilibrium nuclear configurations are often produced at the stage of optical excitation, but they can also be the result of electron tunneling itself, i.e., fast redistribution of charges within the macromolecule. A consistent theoretical description of ultrafast ET requires an explicit consideration of the nuclear subsystem, including its evolution between electron jumps. In this paper, the effect of the multi-timescale nuclear reorganization on ET transitions in macromolecular compounds is studied, and a general theory of ultrafast ET in non-Debye polar environments with a multi-component relaxation function is developed. Particular attention is paid to designing the multidimensional space of nonequilibrium nuclear configurations, as well as constructing the diabatic free energy surfaces for the ET states. The reorganization energies of individual ET transitions, the equilibrium energies of ET states, and the relaxation properties of the environment are used as input data for the theory. The effect of the system-environment interaction on the ET kinetics is discussed, and mechanisms for enhancing the efficiency of charge separation in macromolecular compounds are analyzed.

## 1. Introduction

Intramolecular electron transfer (ET) is an essential component of many biological processes including photosynthesis, cellular respiration, activation of sensory proteins, and others [1,2,3,4,5,6]. In ET reactions, quantum tunneling of an electron between the redox sites within a macromolecule is largely controlled by the environment that creates the necessary preconditions for an electron jump. Both fast fluctuations of solvent polarization and relatively slow and large-scale reorganization of the environment around the redox sites are important in these processes [7,8,9,10].

The role of the environment is even more pronounced in ultrafast photoinduced reactions, where the evolution of the Franck-Condon state is strongly coupled to the medium [11,12,13,14,15]. These reactions often exhibit unusual behavior that does not correspond to the predictions of classical ET theories. For example, photoinduced ET in donor-acceptor compounds may proceed faster than the characteristic timescale of solvent relaxation [16], which indicates the inapplicability of the Zusman model of diffusion along the ET coordinate [17]. Ultrafast reactions are also sensitive to the properties of the pumping pulse [18,19], and often manifest oscillatory kinetics due to vibrational coherence effects [20,21,22].

One of the promising areas of research in the field of photovoltaics and solar energy conversion is the design of macromolecular compounds aimed at efficient photochemical separation of charges [23,24,25]. These compounds commonly involve several redox sites organized in a way that ensures the stabilization of separated charges. This stabilization is achieved by fast and efficient ET from a photosensitizer and primary electron donor to the distant electron-accepting centers, thus preventing loss of energy due to charge recombination. Fast ET transitions in these compounds lead to the formation of nonequilibrium nuclear configurations and strong coupling of the subsequent ET steps to relaxation processes in the environment. To describe these phenomena, in this paper we develop a semiclassical model of multistage intramolecular electron transfer reactions controlled by the medium with a multi-component dielectric relaxation function. Particular attention is paid to the design of the configuration space of the medium, as well as methods for constructing the diabatic free energy surfaces corresponding to the ET states. The study is mainly focused on the role of the polar environment in these processes and does not take into account the ET-active high-frequency ($\hbar \omega \gg k_{B}T$) intramolecular vibrational modes. However, an appropriate extension of the theory is straightforward and can be easily introduced to the model. In what follows we assume that the macromolecule is «rigid» in the course of ultrafast ET, so that we can omit reorganization of slow large-amplitude intramolecular nuclear coordinates and consider only fast dielectric response both from the outer environment and internal low-frequency (classical) degrees of freedom.

Many theoretical approaches describe electron transfer in condensed phase in terms of the energy-gap coordinate—the quantity evaluated by mapping the system’s nuclear degrees of freedom onto the energy gap ΔE between the ET reactant and product states [26,27,28]. Using the ΔE quantity as the reaction coordinate allows one to reduce the dimensionality of the nuclear configuration space, and ultimately admit the one-dimensional picture of ET with the two intersecting electronic surfaces for the reactant and product states in the diabatic presentation. This approach was suggested by Marcus to describe intermolecular electron transfer in polar liquids [29,30], and later was applied to intramolecular processes, including those initiated by optical excitation of reactants [31,32,33,34,35].

When applied to multistage ET reactions involving several redox centers within a macromolecule, the Marcus approach suggests using several reaction coordinates $\Delta E_{k}$, each controlling ET between the two centers. This extension is straightforward and enables one to consider the multistage reaction as a set of elementary ET steps occurring sequentially and/or in parallel. The key problem of this approach is the non-orthogonality of the $\Delta E_{k}$ coordinates [36,37,38]. This non-orthogonality implies some important features of the reaction, for example, the mutual influence of consequent ET steps on each other [39]. However, the multi-$\Delta E_{k}$ approach leads to apparent technical difficulties associated with the use of a non-orthogonal basis. At present, the method has been implemented only for the three-center model systems of the type A${}_{1}$-D-A${}_{2}$ or D-A${}_{1}$-A${}_{2}$, where D is the electron donor, and A${}_{1}$/A${}_{2}$ are the acceptors [38,40].

An alternative approach was proposed by Tang and Norris [41], who introduced two independent solvent coordinates (x,y) to describe ET in a model system involving the initial (reactant) |1〉, intermediate |2〉, and final (product) |3〉 states. This three-component molecular system reproduces the structure of the photosynthetic reaction center, where primary photochemical charge separation proceeds as ultrafast ET from a bacteriochlorophyll special pair dimer P${}^{*}$ (donor) through a bridging accessory bacteriochlorophyll monomer B${}_{L}$ (primary acceptor) to a distant bacteriopheophytin H${}_{L}$ (secondary acceptor). The use of the independent solvent coordinates in this model implies certain assumptions about the medium, namely, its linear response to charge redistribution between the molecular centers. Now, this model is a common framework for the description of ET in molecular triads [42,43,44,45].

Recently a more general theory of solvent-controlled ET in the Debye polar liquids was developed [46], applicable to multistage reactions with an arbitrary number of elementary ET steps. Following Tang and Norris, the configuration space of multistage ET was constructed with the use of independent solvent coordinates $Q_{k}$. The number *K* of the $Q_{k}$ coordinates was shown to be related to the number *N* of the active redox sites according to the equation K=N−1. It was also shown that the ET energy gaps $\Delta E_{k}$ in this model can be calculated by simple linear transformations over the $Q_{k}$ coordinates [46].

In the present paper, the theory developed in ref. [46] is extended to the non-Debye environments, i.e., media characterized by several dielectric relaxation timescales $\tau_{i}$. The aim of this extension is to broaden the scope of the previous theory, and make it applicable to «complex» environments, as one may expect in molecular electronics devices, biomolecules, and other macromolecular compounds [34,47,48]. As a method, we employ «splitting» of the polarization coordinates $Q_{k}$ into the relaxation components, each corresponding to the individual $\tau_{i}$ mode. This approach is similar to that used by Zusman [49], but it is applied here to independent polarization coordinates rather than the energy-gap coordinate $\Delta E_{k}$. In the next section, we formulate the general idea of the method and introduce the corresponding mathematical framework. After that, we consider an illustrative example by applying the general theory to a simple three-center model in a medium with the two-component relaxation function. Finally, the proposed general approach is verified by analyzing the two special cases in which the results of the well-known Najbar/Tachiya and Zusman models of ET are reproduced.

## 2. Results and Discussion

### 2.1. General Formulation of the Theory

We consider a macromolecule containing *N* redox centers and denote $|\varphi_{n}\rangle$ (n=1,⋯,N) the state of the system with the electron localized on the *n*th redox center. We admit here a general case when ET transitions are possible between any $|\varphi_{n}\rangle$ and $|\varphi_{n^{\prime }}\rangle$ states. We introduce the free energy of medium reorganization $\lambda^{\left(nn^{\prime }\right)}$ that quantifies the response of the environment to the shift of the electronic density $|\varphi_{n}\rangle ⇆|\varphi_{n^{\prime }}\rangle$. Since $\lambda^{\left(nn\right)}=0$ and $\lambda^{\left(nn^{\prime }\right)}=\lambda^{\left(n^{\prime }n\right)}$, the $\lambda^{\left(nn^{\prime }\right)}$ values form a square symmetric matrix of the size *N* with zero diagonal elements

(1) $$\hat{\lambda }=\left(\begin{array}{cccc} 0 & \lambda^{\left(12\right)} & \ldots & \lambda^{\left(1N\right)}\\ \lambda^{\left(12\right)} & 0 & \ldots & \lambda^{\left(2N\right)}\\ \vdots & \vdots & \ddots & \vdots \\ \lambda^{\left(1N\right)} & \lambda^{\left(2N\right)} & \ldots & 0 \end{array}\right).$$

The number of independent parameters here is N(N−1)/2.

Complex dynamics of medium polarization in view of multiple redox centers and ET transitions can be considered by extending the method developed in ref [46]. First, we introduce the set of independent polarization coordinates $Q_{k}$, where *k* ranges from 1 to K=N−1. In the linear-response approximation, the diabatic free energy surfaces (FESs) of the $|\varphi_{n}\rangle$ electronic states in the $Q_{k}$ coordinates are written as [46]

(2) $$G^{\left(n\right)}\left(Q\right)=\sum_{k=1}^{K}{\left(Q_{k}-{\overset{ˇ}{Q}}_{k}^{\left(n\right)}\right)}^{2}+{\overset{ˇ}{G}}^{\left(n\right)}.$$

Here ${\overset{ˇ}{Q}}_{k}^{\left(n\right)}$ are the coordinates of the FES minimum (unknown model parameters so far), and ${\overset{ˇ}{G}}^{\left(n\right)}$ is the equilibrium free energy of the *n*th electronic state. The mutual arrangement of the $G^{\left(n\right)}$ surfaces in the Q space is fully determined by the $\hat{\lambda }$ matrix. To show this, one can use the definition of $\lambda^{\left(nn^{\prime }\right)}$

(3) $$\lambda^{\left(nn^{\prime }\right)}\equiv G^{\left(n\right)}\left({\overset{ˇ}{Q}}^{\left(n^{\prime }\right)}\right)-G^{\left(n\right)}\left({\overset{ˇ}{Q}}^{\left(n\right)}\right)$$

and Equation (2) to obtain

(4) $$\lambda^{\left(nn^{\prime }\right)}=\sum_{k=1}^{K}{\left({\overset{ˇ}{Q}}_{k}^{\left(n^{\prime }\right)}-{\overset{ˇ}{Q}}_{k}^{\left(n\right)}\right)}^{2}={\left|D^{\left(nn^{\prime }\right)}\right|}^{2}.$$

It is easy to see from this equation that $D^{\left(nn^{\prime }\right)}$ is a vector in the Q space connecting the $G^{\left(n\right)}$ and $G^{\left(n^{\prime }\right)}$ FES minima [46]. The distance between the ${\overset{ˇ}{Q}}^{\left(n^{\prime }\right)}$ and ${\overset{ˇ}{Q}}^{\left(n\right)}$ points is thus equal to $\sqrt{\lambda^{\left(nn^{\prime }\right)}}$, that is determined only by the free energy of the medium reorganization for the $|\varphi_{n}\rangle \to |\varphi_{n^{\prime }}\rangle$ ET transition.

Dynamic properties of the medium are commonly introduced to the model using the energy-gap autocorrelation function X(t)=〈ΔE(0)ΔE(t)〉/〈ΔE(0)ΔE(0)〉. This function is an experimentally measured quantity and is often approximated by a sum of several exponentials [50,51]

(5) $$X\left(t\right)=\sum_{i=1}^{R}X_{i}\left(t\right)=\sum_{i=1}^{R}x_{i}\exp\left(-t/\tau_{i}\right).$$

The $X_{i}\left(t\right)$ terms in Equation (5) are often referred to as the relaxation components of the medium, with $x_{i}$ and $\tau_{i}$ being the weight and the relaxation timescale of the *i*th component. The $x_{i}$ quantities satisfy the normalization condition $\sum x_{i}=1$. It should be noted here that polar solvents generally reveal 2–3 relaxation components with the $\tau_{i}$ values differing by several times. In mixtures and structured environments, the range of $\tau_{i}$s can be even larger. An early-time X(t) dependence in liquids is often determined using a combination of ultrafast spectroscopic techniques and computational methods (see, e.g., [52]).

Due to the linearity of the medium response, the X(t) splitting into the relaxation components $X\left(t\right)=\left(X_{1}\left(t\right),X_{2}\left(t\right),\cdots ,X_{R}\left(t\right)\right)$ in Equation (5) is expected to be valid, not only for the energy-gap coordinate ΔE (the Zusman method [49]), but for any polarization coordinate as well. Keeping this in mind, we replace the single $Q_{k}$ value with an *R*-dimensional vector as follows

(6) $$Q_{k}\;⟹\;Q_{k}=\left(q_{k1},\;q_{k2},\;q_{k3},\;\cdots ,\;q_{kR}\right),$$

where $q_{ki}$ is the *i*th component of $Q_{k}$ corresponding to the *i*th relaxation mode. The coordinate splitting (6) extends the Q space and produces a *D*-dimensional configuration space, where

(7) $$D=\left(N-1\right)R.$$

**Figure 1:**
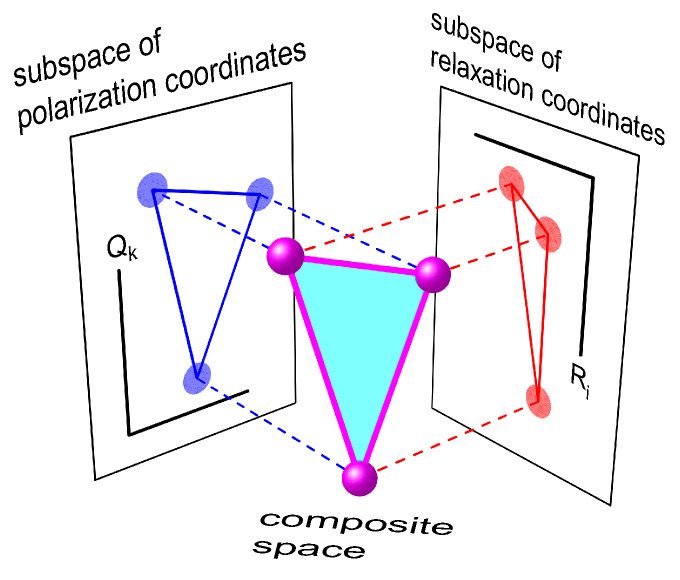
Schematic presentation of subspaces Q and R, as well as the composite space q=Q×R of nuclear configurations of the environment.

By construction, this multidimensional space (denoted in what follows as q) is a direct product of the «polarization» and «relaxation» subspaces, Q and R, with the coordinates $q_{ki}=\left(Q_{k},R_{i}\right)$, where $R_{i}$ (i=1,⋯,R) are the coordinates of R. Figure 1 illustrates this basic idea. We will show later that the splitting method allows one to get rather simple model equations in spite of the expanded dimensionality of the configuration space.

The coordinate splitting (6) is an essentially new element of the theory compared to that presented in ref. [46]. The extended configuration space enables one to describe not only the nonequilibrium polarization of the environment, but also the multi-component relaxation of this polarization to a new equilibrium state. Since the $q_{ki}$ coordinate is directly related to the *i*-th mode of the environment, the system’s relaxation along $q_{ki}$ proceeds with the timescale $\tau_{i}$. This approach allows one to overcome the limitation of the previous theory and develop a more general framework applicable to non-Debye solvents, solvent mixtures, and other environments with complex relaxation functions.

Now we explore the properties of the q space. In the $q_{ki}$ coordinates, Equation (2) becomes

(8) $$G^{\left(n\right)}\left(q\right)=\sum_{k=1}^{N-1}\sum_{i=1}^{R}{\left(q_{ki}-{\overset{ˇ}{q}}_{ki}^{\left(n\right)}\right)}^{2}+{\overset{ˇ}{G}}^{\left(n\right)}.$$

Using (3) and (8), one can find the following expression for the reorganization free energy

(9) $$\lambda^{\left(nn^{\prime }\right)}=\sum_{k,i}{\left({\overset{ˇ}{q}}_{ki}^{\left(n^{\prime }\right)}-{\overset{ˇ}{q}}_{ki}^{\left(n\right)}\right)}^{2}={\left|d^{\left(nn^{\prime }\right)}\right|}^{2}.$$

This expression is similar to Equation (4), but is written for the *D*-dimensional vector $d^{\left(nn^{\prime }\right)}$ in the extended configuration space. Calculating the projection of the $d^{\left(nn^{\prime }\right)}$ vector onto the $R_{i}$ coordinate, $d_{i}^{\left(nn^{\prime }\right)}$, one finds

(10) $$\lambda_{i}^{\left(nn^{\prime }\right)}={\left(d_{i}^{\left(nn^{\prime }\right)}\right)}^{2}.$$

Here $\lambda_{i}^{\left(nn^{\prime }\right)}$ is the part of $\lambda^{\left(nn^{\prime }\right)}$ corresponding to the *i*-th relaxation mode, $\lambda_{i}^{\left(nn^{\prime }\right)}\equiv x_{i}\lambda^{\left(nn^{\prime }\right)}$. Equation (10) can be easily verified by direct summation over the $Q_{k}$ coordinates.

Equations (9) and (10) establish a relation between the known parameters of the model (free energies $\lambda^{\left(nn^{\prime }\right)}$ and relaxation weights $x_{i}$) and unknown quantities ${\overset{ˇ}{q}}_{ki}^{\left(n\right)}$. It follows from Equation (9), that ${\overset{ˇ}{q}}_{ki}^{\left(n\right)}$ can be found using the distances between the FESs minima in the q space, $\left|d^{\left(nn^{\prime }\right)}\right|=\sqrt{\lambda^{\left(nn^{\prime }\right)}}$. This expression allows us to formulate a general scheme for the ${\overset{ˇ}{q}}_{ki}^{\left(n\right)}$ evaluation. As an illustration, we consider a simple three-center model of ET in a medium with two relaxation components. The choice of this model is due to the following reasons: (1) the ${\overset{ˇ}{q}}_{ki}^{\left(n\right)}$ values in this case can be found analytically, (2) the model to be verified for compatibility with earlier results.

### 2.2. Three-Center Molecular System in a Two-Component Environment

**Figure 2:**
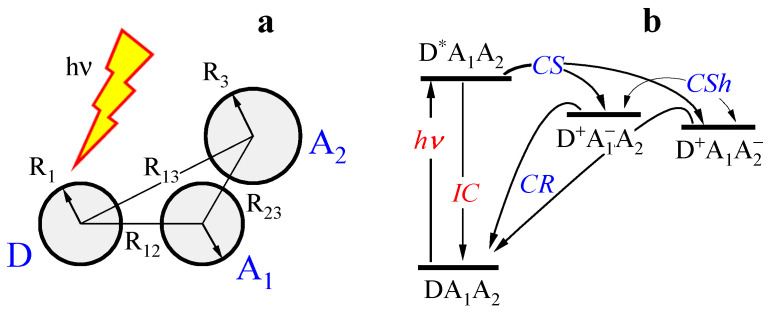
(**a**) Three-center molecular compound of the type DA${}_{1}$A${}_{2}$, where D is the electron-donating, and A${}_{1}$/A${}_{2}$ are the electron-accepting units. The spatial structure of the compound is shown schematically including the effective radii of the units and the center-to-center distances. (**b**) Photochemical processes in this compound. Abbreviations CS, CR, CSh, and IC denote charge separation, charge recombination, charge shift, and internal conversion, respectively.

We consider a molecular triad of the type DA${}_{1}$A${}_{2}$, where D is the electron donor and photosensitizer, and A${}_{1}$/A${}_{2}$ are the electron acceptors. Optical excitation of this compound leads to a population of the locally excited state DA${}_{1}$A${}_{2}$→ D${}^{*}$A${}_{1}$A${}_{2}$, and triggers a series of electronic transitions, including charge separation to the D${}^{+}$A${}_{1}^{-}$A${}_{2}$ and D${}^{+}$A${}_{1}$A${}_{2}^{-}$ states, charge shift between the A${}_{1}$ and A${}_{2}$ units, charge recombination back to the DA${}_{1}$A${}_{2}$ state, and radiationless deactivation of the excited state. The general scheme of the reaction is shown in Figure 2 and involves the following transitions

$$\begin{array}{cc} \; & DA_{1}A_{2}\to D^{*}A_{1}A_{2},\;(\mathrm{optical}\;\mathrm{excitation})\\ \; & D^{*}A_{1}A_{2}\to D^{+}A_{1}^{-}A_{2},\;(\mathrm{charge}\;\mathrm{separation},\;\mathrm{CS}1)\\ \; & D^{*}A_{1}A_{2}\to D^{+}A_{1}A_{2}^{-},\;(\mathrm{charge}\;\mathrm{separation},\;\mathrm{CS}2)\\ \; & D^{+}A_{1}^{-}A_{2}↔D^{+}A_{1}A_{2}^{-},\;(\mathrm{charge}\;\mathrm{shift},\;\mathrm{CSh})\\ \; & D^{+}A_{1}^{-}A_{2}\to DA_{1}A_{2},\;(\mathrm{charge}\;\mathrm{recombination},\;\mathrm{CR}1)\\ \; & D^{+}A_{1}A_{2}^{-}\to DA_{1}A_{2},\;(\mathrm{charge}\;\mathrm{recombination},\;\mathrm{CR}2)\\ \; & D^{*}A_{1}A_{2}\to DA_{1}A_{2},\;(\mathrm{internal}\;\mathrm{conversion},\;\mathrm{IC}). \end{array}$$

It should be noted here that many molecular compounds have the same structure as the redox centers, and can be considered prototype systems. A common and well-known example is the photosynthetic reaction center (RC), where primary charge separation proceeds as a two-step ET involving a BChl special pair dimer (D), an accessory BChl monomer (A${}_{1}$) and a BPh molecule (A${}_{2}$). The spatial arrangement of the D, A${}_{1}$, A${}_{2}$ units in RCs do not facilitate direct electron transfer between D and A${}_{2}$, therefore, the CS${}_{2}$ and CR${}_{2}$ transitions in these compounds are effectively suppressed. This fact, however, does not change the arrangement of the electronic FESs in the q space, since positions of the FESs minima are determined solely by the medium reorganization.

The molecular triad is assumed to interact with a polar environment with the two-component relaxation function X(t). The relaxation parameters of the medium ($x_{1}$, $\tau_{1}$, $x_{2}$, $\tau_{2}$) are considered to be known. Our goal here is to construct a general model of ET in this system taking into account both multiple redox centers and the multi-component relaxation of the medium. It is important to note that this is the simplest model, which has not yet been studied within semiclassical theories. On the one hand, the two-stage ET models in the Debye solvents are known [36,37,38,39,44], and, on the other hand, there are general models of single-step ET in liquids with several relaxation timescales [34,49]. The theory presented here combines both these approaches.

According to Equation (7), the configuration space for a three-center (N=3) molecular system in a two-mode (R=2) medium should minimally involve (N−1)R=4 independent coordinates. It is convenient to represent the 4-dimensional vector q as a 2 × 2 matrix

(11) $$q=\left(\begin{array}{cc} q_{11} & q_{12}\\ q_{21} & q_{22} \end{array}\right).$$

As before, the first index here relates to the polarization coordinate $Q_{k}$, and the second one—to the relaxation component $R_{i}$. We assume that optical excitation does not produce a significant redistribution of the electronic density, so medium reorganization at the stage of excitation can be neglected. According to Equation (9), the positions of the ground-state and the excited-state FESs minima, in this case, coincide, ${\overset{ˇ}{q}}_{ki}^{\left(ex\right)}={\overset{ˇ}{q}}_{ki}^{\left(gr\right)}$. To simplify the description we omit the ground state from consideration and restrict our model to electronic transitions with the charge-transfer character only. The following notation will be used for the ET states

$$|\varphi_{1}\rangle =D^{*}A_{1}A_{2},\;|\varphi_{2}\rangle =D^{+}A_{1}^{-}A_{2},\;|\varphi_{3}\rangle =D^{+}A_{1}A_{2}^{-}.$$

The equilibrium free energies ${\overset{ˇ}{G}}^{\left(1\right)}$, ${\overset{ˇ}{G}}^{\left(2\right)}$, ${\overset{ˇ}{G}}^{\left(3\right)}$, and the reorganization free energies $\lambda^{\left(12\right)}$, $\lambda^{\left(13\right)}$ and $\lambda^{\left(23\right)}$ are considered as known parameters.

We employ Equation (10) to find the FESs minima ${\overset{ˇ}{q}}^{\left(1\right)}$, ${\overset{ˇ}{q}}^{\left(2\right)}$, and ${\overset{ˇ}{q}}^{\left(3\right)}$. Since this equation deals only with relative distances $d_{i}^{\left(nn^{\prime }\right)}$, the ${\overset{ˇ}{q}}^{\left(n\right)}$ points are invariant under the shift and rotation in the *q*-space as a whole. This allows us to choose ${\overset{ˇ}{q}}^{\left(1\right)}$ arbitrarily, for example, by placing it into the origin of the coordinate system

(12) $${\overset{ˇ}{q}}^{\left(1\right)}=\left(\begin{array}{cc} 0 & 0\\ 0 & 0 \end{array}\right).$$

Now, to find ${\overset{ˇ}{q}}^{\left(2\right)}$, we use the known distance to the ${\overset{ˇ}{q}}^{\left(1\right)}$ point, which is equal to $\sqrt{\lambda^{\left(12\right)}}$ according to Equation (9). At this step, it is enough to use only one of the two polarization coordinates, say $Q_{1}$. From Equation (10) one finds $\left|{\overset{ˇ}{q}}_{11}^{\left(1\right)}-{\overset{ˇ}{q}}_{11}^{\left(2\right)}\right|=\sqrt{\lambda^{\left(12\right)}x_{1}}$ and $\left|{\overset{ˇ}{q}}_{12}^{\left(1\right)}-{\overset{ˇ}{q}}_{12}^{\left(2\right)}\right|=\sqrt{\lambda^{\left(12\right)}x_{2}}$. This gives the following result

(13) $${\overset{ˇ}{q}}^{\left(2\right)}=\left(\begin{array}{cc} \sqrt{\lambda^{\left(12\right)}x_{1}} & \sqrt{\lambda^{\left(12\right)}x_{2}}\\ 0 & 0 \end{array}\right).$$

Similar algorithm can then be applied to ${\overset{ˇ}{q}}^{\left(3\right)}$. This point should be arranged in such a way that distances from ${\overset{ˇ}{q}}^{\left(3\right)}$ to ${\overset{ˇ}{q}}^{\left(1\right)}$ and ${\overset{ˇ}{q}}^{\left(2\right)}$ satisfy Equation (10). To fulfill these conditions, both polarization coordinates $Q_{1}$ and $Q_{2}$ are needed. Finally, one gets

(14) $${\overset{ˇ}{q}}^{\left(3\right)}=\left(\begin{array}{cc} \sqrt{\lambda^{\left(13\right)}x_{1}}\cos\theta & \sqrt{\lambda^{\left(13\right)}x_{2}}\cos\theta \\ \sqrt{\lambda^{\left(13\right)}x_{1}}\sin\theta & \sqrt{\lambda^{\left(13\right)}x_{2}}\sin\theta \end{array}\right),$$

where

(15) $$\cos\theta =\frac{\lambda^{\left(12\right)}+\lambda^{\left(13\right)}-\lambda^{\left(23\right)}}{2\sqrt{\lambda^{\left(12\right)}\lambda^{\left(13\right)}}}.$$

Equations (12)–(14) can be easily verified by direct evaluation of the $d_{i}^{\left(12\right)}$, $d_{i}^{\left(13\right)}$, and $d_{i}^{\left(23\right)}$ components, and comparing them with Equation (10).

The $G^{\left(n\right)}\left(q\right)$ FESs, constructed using Equations (8) and (12)–(15), completely determine the energy barriers for all ET steps. As an example we consider the $|\varphi_{1}\rangle \to |\varphi_{2}\rangle$ transition, i.e., transfer of an electron from D${}^{*}$ to A${}_{1}$. The ET is possible only if the reactant and product energies are equal, $G^{\left(1\right)}\left(q\right)=G^{\left(2\right)}\left(q\right)$. The solution of this equation is a hyperplane in the 4-dimensional space

(16) $$q_{11}\sqrt{\lambda^{\left(12\right)}x_{1}}+q_{12}\sqrt{\lambda^{\left(12\right)}x_{2}}=\lambda^{\left(12\right)}+{\overset{ˇ}{G}}^{\left(2\right)}-{\overset{ˇ}{G}}^{\left(1\right)},$$

that describes the intersection of the two parabolic FESs. The section itself is a three-dimensional paraboloid, and the minimum value on this surface is the saddle point between the reactant and product FESs (we denote it as $q^{♯}$). The height $H_{12}^{♯}$ of the energy barrier between the equilibrium states then can be calculated using the $q^{♯}$ value. This parameter is very important for the analysis of thermal (quasi-equilibrium) reactions and included many expressions for the ET rate constants [26,28,33]. At the same time, the reaction pathway in the case of nonequilibrium ET may not be limited to the saddle point, especially in ultrafast processes, where the reaction flux deviates from the saddle point significantly [53]. The effective energy barrier in ultrafast ET may therefore differ from $H_{12}^{♯}$.

These peculiarities of nonequilibrium ET can be taken into account by considering the motion of the wave packets on the corresponding FESs along with ET transitions. We introduce the time-dependent density function $\rho_{n}\left(q,t\right)$ for the *n*th ET state of the system. According to the Zusman method [17], the $q_{ki}$ dynamics can be considered as diffusion, and chemical transformations as reversible quantum transitions localized at the intersection regions of the reactant and product FESs. Applying this method, one obtains the following equation for $\rho_{1}\left(q,t\right)$

(17) $$\begin{array}{cc} \frac{\partial \rho_{1}\left(q,t\right)}{\partial t} & ={\hat{L}}_{1}^{\left(1\right)}\rho_{1}+{\hat{L}}_{2}^{\left(1\right)}\rho_{1}+\\ & +\frac{2\pi V_{12}^{2}}{\hbar }\;\delta \;\left(G^{\left(1\right)}-G^{\left(2\right)}\right)\left(\rho_{2}-\rho_{1}\right)+\frac{2\pi V_{13}^{2}}{\hbar }\;\delta \;\left(G^{\left(1\right)}-G^{\left(3\right)}\right)\left(\rho_{3}-\rho_{1}\right). \end{array}$$

The last two terms on the right-hand side describe the ET transitions $|\varphi_{1}\rangle ↔|\varphi_{2}\rangle$ and $|\varphi_{1}\rangle ↔|\varphi_{3}\rangle$ in the nonadiabatic limit. $V_{12}$ and $V_{13}$ are the coupling energies between the corresponding electronic states, ${\hat{L}}_{1}^{\left(n\right)}$ and ${\hat{L}}_{2}^{\left(n\right)}$ are the Smoluchowski operators of diffusion on the $G^{\left(n\right)}$ FES. It is important that the diffusion coefficients along the ($q_{11}$, $q_{21}$) coordinates and along the ($q_{12}$, $q_{22}$) coordinates are different, since these coordinates are associated with different timescales of polarization relaxation ($\tau_{1}$ and $\tau_{2}$)

(18) $${\hat{L}}_{i}^{\left(n\right)}=\frac{1}{\tau_{i}}\sum_{k=1}^{2}\left(1+\left(q_{ki}-{\overset{ˇ}{q}}_{ki}^{\left(n\right)}\right)\frac{\partial }{\partial q_{ki}}+k_{B}T\frac{\partial^{2}}{\partial q_{ki}^{2}}\right).$$

Similar kinetic equations can be written for the $\rho_{2}\left(q,t\right)$ and $\rho_{3}\left(q,t\right)$ densities, they have the same structure as Equation (17). The resulting set of equations accomplishes the formulation of the model. This set can also be supplemented with the initial conditions, which usually reflect the conditions of the initial state formation. In the case of activated ET from the $|\varphi_{1}\rangle$ state, the initial distribution on the $G^{\left(1\right)}$ FES is often assumed to be thermodynamically equilibrium

(19) $$\rho_{1}\left(q,t=0\right)=\prod_{k,i}\frac{1}{\sqrt{2\pi k_{B}T}}\exp\left(-\frac{{\left(q_{ki}-{\overset{ˇ}{q}}_{ki}^{\left(n\right)}\right)}^{2}}{2k_{B}T}\right).$$

In ultrafast photoreactions, on the contrary, the initial distribution in the excited state is generally nonequilibrium and depends both on the energetic parameters of the system and spectral characteristics of the pumping pulse (see, e.g., [54]).

The proposed theory can be used as a framework for numerical simulations and analysis of experimental data on ultrafast ET in macromolecules. The theory can be linked to experiments, for example, by calculating the populations of the electronic states and comparing them with the observed ET kinetics. In the present model, the $|\varphi_{n}\rangle$ state population is evaluated as the integral of the density function over the entire configuration space

(20) $$P_{n}\left(t\right)=\int dq\;\rho_{n}\left(q,t\right).$$

Another important quantity of ultrafast ET is the quantum yield of charge separation $Y_{\mathrm{CS}}$, which is commonly identified with the total population of charge-transfer states at the time *T* when relaxation processes are over [55,56]

(21) $$Y_{\mathrm{CS}}=P_{2}\left(T\right)+P_{3}\left(T\right),\;\mathrm{where}\;T=5\max\left\{\tau_{1},\tau_{2}\right\}.$$

It is important to note here, that the $\rho_{n}\left(q,t\right)$ functions can also be used for simulations of luminescent properties of the system in the course of ET. Recently, specific computational methods have been developed for simulations of the reaction kinetics and transient absorption/emission spectra of nonequilibrium macromolecules [46,57,58], but within simpler models that do not take into account complex dynamics of medium relaxation. Similar algorithms can also be developed for the generalized theory presented here, but these results will be reported elsewhere.

## 3. Methods and Materials

### Verification of the Theory

The proposed theory can be verified by comparing it with earlier models, especially those that can be considered as special cases with respect to the present general approach. Hereafter, the following well-known frameworks are used for this verification: (1) the Najbar/Tachiya theory of two-stage ET in the Debye polar solvent [37], (2) the Zusman theory of ET in solvents with a two-component relaxation function [49]. Both these theories are special cases: the Najbar/Tachiya theory was developed for the three-center molecular compounds (N=3), but only in a single-component solvent (R=1); the Zusman theory is applicable to the multi-component environments (any *R*), but involves ET between only two redox centers (N=2) and thus does not provide appropriate description of multistage reactions. The difference between these two models can also be clarified by comparing their configuration spaces. Both spaces are two-dimensional, but their nature is completely different. In the Najbar/Tachiya model, the free energy surfaces are constructed as functions of the polarization coordinates, while the Zusman model employs the relaxation coordinates. The two models thus operate in different subspaces as shown in Figure 1. Since the theory presented in this paper is a generalization of the two approaches, we expect the new theory to reproduce the results of the previous ones in domains of their applicability according to the correspondence principle.

To demonstrate the correspondence to the Najbar/Tachiya model, we set $\tau_{1}=\tau_{2}$ and eliminate the relaxation subspace R from consideration by projecting the composite space q onto the subspace Q

(22) $$Q_{k}=\left|Q_{k}\right|=\sqrt{\sum_{i}q_{ki}^{2}}.$$

This R-space folding gives the following coordinates of the FES minima

(23) $${\overset{ˇ}{Q}}^{\left(1\right)}=\left(\begin{array}{c} 0\\ 0 \end{array}\right),\;{\overset{ˇ}{Q}}^{\left(2\right)}=\left(\begin{array}{c} \sqrt{\lambda^{\left(12\right)}}\\ 0 \end{array}\right),\;{\overset{ˇ}{Q}}^{\left(3\right)}=\left(\begin{array}{c} \sqrt{\lambda^{\left(13\right)}}\cos\theta \\ \sqrt{\lambda^{\left(13\right)}}\sin\theta \end{array}\right).$$

The $G^{\left(n\right)}$ FESs in the $\left(Q_{1},Q_{2}\right)$ coordinates take the form of Equations (2). This result reproduces the Najbar/Tachya model [37], where two-stage ET is considered in terms of two-dimensional parabolic surfaces. It should also be noted that two-dimensional presentation of FESs in the form (2), (28) has been repeatedly used earlier (see, for example, [34,45]).

Consider the 3-center molecular system in more detail, assuming the electronic densities on D, A${}_{1}$, A${}_{2}$ to be described by the effective radii $r_{1}$, $r_{2}$, $r_{3}$, and the distances $r_{12}$, $r_{13}$, $r_{23}$ between the centers. This simple model is shown in Figure 2a, and allows us to estimate the system’s energetics using the Marcus formula for the reorganization energy in a continuous dielectric medium

(24) $$\lambda_{ij}=\frac{c_{p}e^{2}}{2}\left(\frac{1}{r_{i}}+\frac{1}{r_{j}}-\frac{2}{r_{ij}}\right).$$

Here *e* is the charge of an electron, $c_{p}=\epsilon_{o}^{-1}-\epsilon_{s}^{-1}$ is the Pekar factor, $r_{i}$ and $r_{j}$ are the effective radii of the donating and accepting sites, and $r_{ij}$ is the center-to-center distance between them. We adopt here the following model parameters that provide relatively high yields of charge separation [44]

(25) $$r_{1}=r_{2}=4,\;r_{3}=8,\;r_{12}=10.4,\;r_{23}=12,\;r_{13}=19.4\;(\mathrm{in}\;\mathrm{angstroms}).$$

As a solvent, we take highly polar acetonitrile (ACN, $\varepsilon_{\infty }=1.806$, $\varepsilon_{0}=36.64$) with a two-component dielectric relaxation [50,51]. The X(t) function parameters in ACN are: $x_{1}=0.686$, $\tau_{1}=0.089$ ps, $x_{2}=0.314$, $\tau_{2}=0.63$ ps.

Using these model parameters one can estimate the reorganization energies from Equation (24)

(26) $$\lambda^{\left(12\right)}=1.17\;\mathrm{eV},\;\lambda^{\left(13\right)}=1.03\;\mathrm{eV},\;\lambda^{\left(23\right)}=0.79\;\mathrm{eV},\;\theta =50^{\circ }.$$

and calculate the diabatic FESs minima from Equations (12)–(15)

(27) $${\overset{ˇ}{q}}^{\left(1\right)}=\left(\begin{array}{cc} 0 & 0\\ 0 & 0 \end{array}\right),\;{\overset{ˇ}{q}}^{\left(2\right)}=\left(\begin{array}{cc} 0.894 & 0.605\\ 0 & 0 \end{array}\right),\;{\overset{ˇ}{q}}^{\left(3\right)}=\left(\begin{array}{cc} 0.540 & 0.365\\ 0.645 & 0.436 \end{array}\right).$$

With known values ${\overset{ˇ}{G}}^{\left(1\right)}=0$, ${\overset{ˇ}{G}}^{\left(2\right)}=-0.2$ eV and ${\overset{ˇ}{G}}^{\left(3\right)}=-0.1$ eV we have a set of diabatic FESs ${\overset{ˇ}{G}}^{\left(n\right)}$ in the 4-dimensional composite space q.

To illustrate the *R*-space «folding» (Equation (22)), we calculate the projections of the ${\overset{ˇ}{q}}^{\left(n\right)}$ points into the *Q* subspace. They are

(28) $${\overset{ˇ}{Q}}^{\left(1\right)}=\left(\begin{array}{c} 0\\ 0 \end{array}\right),\;{\overset{ˇ}{Q}}^{\left(2\right)}=\left(\begin{array}{c} 1.080\\ 0 \end{array}\right),\;{\overset{ˇ}{Q}}^{\left(3\right)}=\left(\begin{array}{c} 0.652\\ 0.779 \end{array}\right).$$

**Figure 3:**
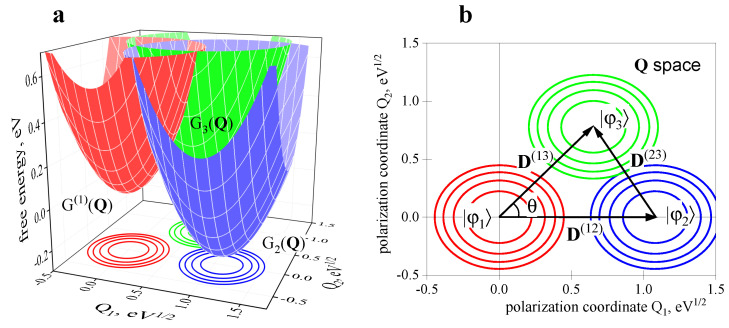
(**a**) Free energy surfaces of the ET states $|\varphi_{1}\rangle =D^{*}A_{1}A_{2}$, $|\varphi_{2}\rangle =D^{+}A_{1}^{-}A_{2}$ and $|\varphi_{3}\rangle =D^{+}A_{1}A_{2}^{-}$ as functions of the polarization coordinates. These curves are constructed by projection of the 4-dimensional $G^{\left(n\right)}\left(q\right)$ surfaces into the 2-dimensional Q subspace. The values of the model parameters are indicated in the text. (**b**) FESs minima on the $\left(Q_{1},Q_{2}\right)$ plane. The displacement vectors $D^{\left(nn^{\prime }\right)}$ and the θ angle are also shown.

Figure 3 shows parabolic FESs calculated using Equation (28). Panel b) also shows the displacement vectors $D^{\left(nn^{\prime }\right)}$ and the angle θ between the directions of the $|\varphi_{1}\rangle \to |\varphi_{2}\rangle$ and $|\varphi_{1}\rangle \to |\varphi_{3}\rangle$ transitions. The standard one-dimensional ET picture of two intersecting parabolic curves can be obtained by cutting the surfaces in Figure 3a by vertical planes passing through the minima of the reactant and product FESs.

Now we check the present theory for its correspondence to the Zusman model of ET in solvents with two relaxation timescales [49]. To do this, we eliminate the polarization subspace Q by projecting the q space onto the relaxation subspace R. This projection is done as follows

(29) $$R_{i}=\left|R_{i}\right|=\sqrt{\sum_{k}q_{ki}^{2}}.$$

From Equations (12)–(14) one gets

(30) $${\overset{ˇ}{R}}^{\left(1\right)}=\left(\begin{array}{c} 0\\ 0 \end{array}\right),\;{\overset{ˇ}{R}}^{\left(2\right)}=\left(\begin{array}{c} \sqrt{\lambda^{\left(12\right)}x_{1}}\\ \sqrt{\lambda^{\left(12\right)}x_{2}} \end{array}\right),\;{\overset{ˇ}{R}}^{\left(3\right)}=\left(\begin{array}{c} \sqrt{\lambda^{\left(13\right)}x_{1}}\\ \sqrt{\lambda^{\left(13\right)}x_{2}} \end{array}\right).$$

In the Zusman model, the FESs displacements in relaxation coordinate $R_{i}$ are proportional to the corresponding weight factors $x_{i}$ [49]. The same result is valid for our model, where the ${\overset{ˇ}{R}}_{i}^{\left(n\right)}$ coordinates are located along a straight line given by the equation $R_{2}=\sqrt{x_{2}/x_{1}}R_{1}$ (see Equation (31)). Such an arrangement provides the required ratio of the relaxation components for all ET transitions in the system. Equation (17) for the system evolution is transformed into the Smoluchowski equation of diffusion in a two-dimensional parabolic potential. Diffusion coefficients along the $R_{1}$ and $R_{2}$ coordinates, however, are different: $D_{1}=k_{B}T/\tau_{1}$ and $D_{2}=k_{B}T/\tau_{2}$. These equations reproduce the results of ref [49] as well.

Applying the Q-folding transformation to our model system (Equation (27)) we find the FESs minima in the R subspace

(31) $${\overset{ˇ}{R}}^{\left(1\right)}=\left(\begin{array}{c} 0\\ 0 \end{array}\right),\;{\overset{ˇ}{R}}^{\left(2\right)}=\left(\begin{array}{c} 0.894\\ 0.605 \end{array}\right),\;{\overset{ˇ}{R}}^{\left(3\right)}=\left(\begin{array}{c} 0.841\\ 0.569 \end{array}\right).$$

**Figure 4:**
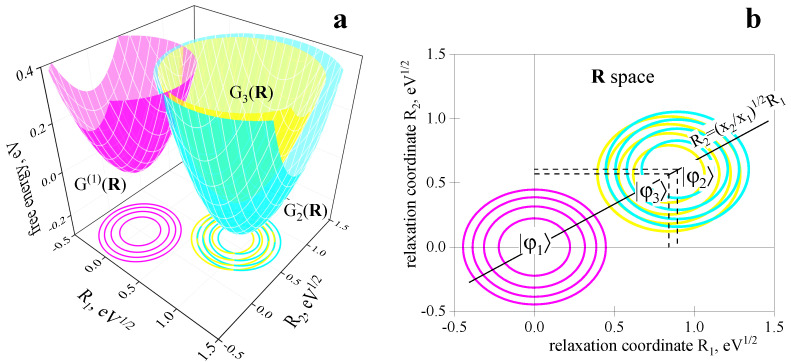
(**a**) Free energy surfaces of the ET states as functions of the relaxation coordinates $R_{1}$ and $R_{2}$. The surfaces are calculated by orthogonal projection from the q space onto the. The $x_{1}$ and $x_{2}$ parameters correspond to acetonitrile (given in the text), other parameters are the same as in Figure 3. (**b**) Arrangement of the FESs minima on the $\left(R_{1},\;R_{2}\right)$ plane: the ${\overset{ˇ}{R}}^{\left(n\right)}$ points are located on the straight line given by the equation $R_{2}/R_{1}=\sqrt{x_{2}/x_{1}}$.

The $G^{\left(n\right)}$ curves on the $\left(R_{1},R_{2}\right)$ plane are pictured in Figure 4.

## 4. Conclusions

Many studies of photoinduced ET in macromolecules are related to the prospects for their use as components of molecular electronics devices, in particular, solar cells, organic light-emitting diodes, optical sensors, switches, and others. Ultrafast photochemical reactions on the femtosecond timescale are often accompanied by the nonequilibrium states of the reactants and the environment. Multistage photoinduced ET in non-Debye environments, such as polymer mixtures, protein matrices, nanoaggregates, etc., is of particular interest. To describe the nonequilibrium effects in these reactions, we propose a method based on the splitting of the independent polarization coordinates into the relaxation components. The method can be considered as a generalization of the two well-known approaches, one of which is used to describe multistage processes, and the other, to account for the multi-component relaxation of the environment. In this study, the properties of the extended space are studied, and the relationship between the reorganization free energy, the medium relaxation parameters, and the arrangement of the diabatic FESs in the extended configuration space is established. The proposed general approach is applied to a three-center molecular system in a medium with a two-component relaxation function. The algorithm for the diabatic FESs construction is described in detail, and a set of kinetic equations for the electronic density functions is specified. The general approach is verified by demonstrating its correspondence to the well-known Najbar/Tachiya and Zusman models.

## Data Availability

Not applicable.
